# An online alcohol and risky sex prevention program for college students studying abroad: study protocol for a randomized controlled trial

**DOI:** 10.1186/s13722-019-0162-4

**Published:** 2019-08-20

**Authors:** Eric R. Pedersen, Elizabeth J. D’Amico, Joseph W. LaBrie, Coreen Farris, David J. Klein, Beth Ann Griffin

**Affiliations:** 1 RAND Corporation, 1776 Main Street, PO Box 2138, Santa Monica, CA 90407-2138 USA; 2 Department of Psychology, Loyola Marymount University, 1 LMU Drive, Los Angeles, CA 90045 USA; 3 RAND Corporation, 4570 Fifth Ave, Ste. #600, Pittsburgh, PA 15213 USA; 4 RAND Corporation, 1200 South Hayes Street, Arlington, VA 22202 USA

**Keywords:** Alcohol, Sexual violence, Intervention, Online, Study abroad, Young adults

## Abstract

**Background:**

This study protocol describes a proposed randomized controlled trial that builds upon a successful pilot intervention study to address problematic and dangerous drinking among young adult college students studying abroad in foreign environments. Despite universities and colleges citing alcohol misuse as the most concerning issue for their students abroad, most institutions offer no empirically-based prevention efforts tailored to this at-risk population. The proposed intervention attempts to fill a major gap for the nearly 333,000 students completing study abroad programs each year by using empirically-based and theoretically-informed risk and protective factors to correct misperceived peer drinking norms and promote cultural engagement abroad. In addition to preventing heavy and problematic drinking, the intervention seeks to prevent risky sexual behaviors (e.g., sex without a condom) and experience of sexual violence victimization, which are strikingly common among study abroad students and have the potential for lasting physical and psychological effects upon return home.

**Methods/design:**

We will conduct a randomized controlled trial of an intervention with a sample of 1200 college students studying abroad from approximately 50 US universities and colleges. The brief, online intervention is text and video based and contains evidence-based components of personalized normative feedback to correct students’ misperceived drinking norms, content to promote engagement with the cultural experience abroad and address difficulties adjusting to life in the foreign environment, and tips and strategies to prevent risky sexual behaviors and sexual violence victimization abroad. Participants will complete online surveys at five time points (predeparture, first month abroad, last month abroad, 1-month post-return, and 3-months post-return) to assess for intervention effects on drinking behavior, drinking consequences, risky sex, and sexual violence outcomes. We will examine whether the mechanisms targeted by the intervention (changes in perceived norms, engagement in the cultural experience abroad) serve as mediators of intervention efficacy.

**Discussion:**

The proposed study has the potential to fill an important gap in the research literature and provide empirical support for an online accessible, brief, and targeted approach that can easily be distributed to study abroad students to help prevent heavy alcohol use and sexual risk abroad.

*Trial registration* ClinicalTrials.gov Identifier NCT03928067

## Background

The number of young adult students taking advantage of opportunities to study abroad during college is growing. Approximately 1 in 10 American undergraduate college students study abroad before graduating, with nearly 333,000 students completing study abroad experiences in the 2016/2017 academic year [1]. This represents a 120% increase over the past 15 years. The personal, cultural, and academic benefits of study abroad are many and include increased global perspectives, enhanced self-esteem, preparation for international careers, respect for other cultures, and academic success [2–8].

### Study abroad students are at-risk for heavy drinking and negative sexual consequences

Despite these benefits, American study abroad students represent a large and diverse population at-risk for increased and problematic drinking. Students more than double their weekly alcohol use while abroad and both pre-abroad inexperienced drinkers and the heaviest drinkers abroad return home drinking at higher levels than before they left [9–11]. A substantial portion of students face negative alcohol-related consequences while abroad [9, 10]. For example, in one study, more than one-third of study abroad students reported taking risks or doing impulsive things while drinking they later regretted [9], and in another study, at least 1 in 10 students reported drinking to the point of passing out or blacking out, missing classes, finding themselves in dangerous situations, or experiencing alcohol-related injuries [10]. The novelty of the abroad context creates a wider range of abroad-specific consequences than are present on campus (e.g., offending host families, losing passports, disrupted travel plans) [9]. In addition, consequences may be exacerbated abroad or develop into long-term problems due to limited access to resources and familiar coping strategies (e.g., being far from friends/family; being unfamiliar with local healthcare locations or law enforcement policies). University administrators and personnel working with study abroad students also report substantial concerns with their students’ drinking behavior abroad [12–15].

In addition to heavy drinking, students are also exposed to a number of sexual risks while abroad, including risky sexual behaviors (e.g., unprotected sex with multiple partners) and sexual violence (ranging from non-consensual sexual contact to attempted and completed sexual assault by force) [16]; many of these risks are amplified by heavy drinking. A well-established line of research with college students on campus indicates that heavy alcohol use increases risky sexual behavior, and is associated with increased risk for sexual assault victimization and perpetration among both male and female students [17–20]. Increases in heavy drinking within a novel environment alone may place students at risk for sexual violence victimization. Back on campus, at least half of sexual violence incidents on campus involve victim and/or perpetrator drinking; most often both are intoxicated [19, 21]. Heavy drinkers are most at-risk, but even light or non-drinkers that engage in heavy drinking during a particular situation (such as during an abroad trip) are at increased sexual risk [22]. Thus, the nature of heavy drinking abroad, coupled with unique risks found in the abroad environment, likely contribute to greater sexual risk abroad.

The literature on sexual risk abroad is only emerging, however, small cross sectional studies from single universities have shown that between 25 and 50% of male and female students report risky sexual behavior [9, 23, 24], upwards of 75% of female students report some form of sexual victimization while abroad [23–26], and female students are at three times higher risk for attempted sexual assault and five times higher risk for completed sexual assault abroad compared to their risk back home on campus [25], where rates are already high (upwards of 1 in 5 female students) [27]. Men are also not immune to sexual victimization abroad; in one study nearly 1 in 10 reported pressure to have sex with someone while intoxicated [9]. These events can ruin study abroad experiences and lead to lasting physical and psychological effects (e.g., sexually transmitted infections, pregnancy, trauma).

### The need for evidence-based prevention programs specific to study abroad risk

A review of study abroad drinking revealed that while there is ample evidence showing that drinking in study abroad contexts is more problematic than domestic on-campus drinking, there has been little attention or targeted interventions aimed at this high-risk environment [28]. In addition, content analysis of 753 US study abroad program websites revealed that institutions provide limited information about alcohol use and sexual risk on their websites [29]. Furthermore, although study abroad program directors report that they discuss alcohol and associated sexual risks with their students prior to abroad [30], the majority of students report that they do not receive any predeparture alcohol or sexual risk programming [31]. Thus, a program designed to target this large at-risk group and the unique risk factors faced abroad has the potential to fill an important gap and produce widespread and lasting physical and/or psychological benefits. Though most college campuses provide all students alcohol programs and sexual violence prevention programs, the nature and extent of heavy drinking, risky sex, and sexual victimization abroad are very different than on campus, and programs geared toward all students (and in particular, those on campus) may not target the specific risks experienced by students when the go abroad. Increased drinking rates combined with unfamiliar surroundings, impaired judgment around the prudence of certain behaviors in the novel cultural environment, and limited comprehension of the nuances of local culture may increase the potential for negative alcohol and sexual outcomes. Thus, there is a need for prevention programs specifically targeted toward study abroad students and their unique risks in the novel environment.

### Risk and protective factors for study abroad students

#### Normative drinking misperceptions

Incorrect perceptions of drinking abroad are a major factor contributing to increased and problematic drinking while studying abroad. Within the study abroad environment, perceived norms of study abroad peers are robustly associated with heavy drinking abroad and predict increases in drinking behavior from predeparture to during the abroad experience [9, 32, 33]. In addition to perceptions of other US student peer drinking while abroad, perceptions of the normative drinking of native young adults in one’s host country are uniquely associated with student drinking [32], such that if a student believes local people are heavier drinkers, he or she is, in turn, likely to drink more heavily as well. This is true even when controlling for per capita alcohol consumption in the students’ host countries [32], suggesting that perceptions of what local people are doing are more influential on personal drinking than the actual drinking levels of the host country.

Targeting misperceptions of peer alcohol use through personalized normative feedback (PNF) interventions can correct misperceptions and prevent heavy drinking during high-risk events. Correcting misperceptions of peer drinking norms through PNF has become one of the most prominent strategies for addressing excessive alcohol use among college students [34–38]. Interventions based solely on PNF have demonstrated small to large effect sizes [39–41], with changes in perceptions of peer behavior mediating intervention effects [42–45]. Even in interventions that include several components in addition to PNF, the effects are generally mediated by augmented normative perceptions rather than variables associated with the other components typical to brief interventions (e.g., weighing pros and cons of changing behavior) [46–48]. Most of this research has focused on reducing pre-existing drinking patterns; however, in recent work, PNF interventions have been adapted to proactively prevent drinking during specific future events known to represent heightened risk, such as 21st birthday and spring break trips [41, 49–51].

In our pilot work with a sample of 343 study abroad students from one university, we found that providing PNF focused on salient and proximal reference groups (i.e., study abroad peers; country-specific young adults native to one’s host country) prior to their departure helped students form more accurate beliefs about drinking within their host country and prevented escalation of drinking while abroad [52]. However, we also found that heavier drinking students that received PNF alone failed to report significantly less drinking during abroad than those heavy drinkers not receiving PNF. This may be because students expect to drink more while abroad and believe alcohol will be a central part of their abroad experience [9]. Helping students navigate this unique environment could reduce risk and consequences. In addition, a key aspect of PNF is that, after learning the actual norms for their environment, participants confirm the veracity of these new norms themselves by observing the behavior of peers and local people post intervention. However, this critical norm confirmation step is missed among students abroad who spend little time engaging their new cultural environment (e.g., spending most of one’s time socializing with other American students) because such students avoid the local sociocultural environment where these cross-cultural drinking norms would be visible. Thus, study abroad students may require an additional innovative approach that not only addresses the perceived norms that can contribute to increased drinking while abroad, but also addresses the importance of understanding one’s host country and the study abroad context. Doing so can help students navigate this new environment more successfully and therefore be less likely to drink heavily.

#### Sojourner adjustment

Poor adjustment into the foreign environment and limited engagement with the local culture represent additional risk factors for heavy drinking while abroad. Theories of acculturation [53, 54] and, more specifically, sojourner adjustment (i.e., the sociocultural and psychological adjustment of relatively short-term visitors to new cultures) [55] posit that immigrants and students who attempt to integrate or assimilate more fully into their new culture are at the least risk for sociocultural and psychological adjustment difficulties [53, 54, 56–60]. Research and theory also suggest that if young people transition to a temporary novel risky environment (e.g., spring break, Mardi Gras in New Orleans) and they do not feel connected to their environment, they may view their time as a temporary reprieve from real life (i.e., a “backspace”), transgress drinking and sexual norms, act in ways inconsistent with their personalities, and, thus, be more likely to engage in risky behaviors such as heavy drinking and risky sex [61–64].

Our research with American study abroad students suggests that those most at-risk for heavy drinking and consequences are those who separate themselves from the host environment (i.e., placing more emphasis on the home/US culture), those who perceive the abroad culture as very different than their home culture, those who spend more time with other Americans while abroad, those who feel out of place being away from home, and those who experience anxiety about interacting in the foreign environment [9, 32]. Other work has confirmed that this *negative* sojourner adjustment is associated with greater risk for heavy drinking and problems abroad [65, 66]. Conversely, we have also found that *positive* sojourner adjustment (i.e., quality/quantity of time with local people, active engagement with cultural experiences and events, foreign language development/use, identification as a member of the host culture) protects students from heavy alcohol use and problems [10]. Thus, promoting cultural engagement and helping with adjustment/transition to life in a foreign environment may prevent incidences of problematic drinking abroad [2, 67–69]. Positive sojourner adjustment can relate to taking advantage of cultural learning experiences by participating in local customs and spending time with local people, rather than focusing on drinking-centered social experiences with other American students. Culturally-engaging activities may also serve as healthy alternatives and provide a means of achieving social and recreational goals without drinking [70], potentially reducing motivation to drink for both social and coping reasons and, therefore, reducing alcohol use and consequences while abroad. Importantly, preventing heavy drinking patterns from forming abroad may also prevent continued heavy drinking once students return home.

#### Targeting both normative drinking misperceptions and sojourner adjustment

Our work has shown that study abroad students who greatly overestimated drinking behavior and who reported negative sojourner adjustment had the highest risk of drinking and consequences [32]. It was also evident from our pilot study that providing PNF alone, which works well for students back on campus, was not a sufficient stand-alone intervention for study abroad students—an established heavy drinking group at risk for multiple negative consequences. Misperceived norms back on campus are based on years of observation of peers (Social Learning Theory [71]; e.g., noticing the few heavy drinkers at a party and falsely believing these heavy drinkers are the majority) and the perpetuation of college heavy drinking stereotypes (e.g., movies such as *Animal House* and *Old School*; discussions with friends about the parties that occurred). Yet study abroad students are entering a new environment with little to no context to make predictions about what drinking norms may look like in the foreign country (both by their study abroad peers and by local young adults). The promotion of cultural engagement in addition to PNF can help students better understand ways to engage in this new environment without the exclusive company of their American peers, affording valuable opportunities to observe that their perceptions of drinking may have been inaccurate. Our pilot work showed that students provided with sojourner adjustment feedback (SAF) in addition to PNF prior to departure abroad experienced significantly fewer alcohol-related consequences abroad compared to students in a control condition, with the greatest benefits for the most at-risk students—those reporting heavier consequences at predeparture [52].

#### Other risk factors

Risk factors inherent to the abroad environment are also present and will likely impact any intervention effects on programming seeking to prevent heavy drinking and problems abroad. Many students may be going abroad before they turn 21 years old, which is the legal age to purchase and consume alcohol in the United States, but in nearly all countries that students travel to, the drinking age is lower. Thus, students may be studying abroad in cultures where it is now legal for them to access alcohol and research shows that those under 21 are at greater risk for increased drinking abroad [11, 72]. Moreover, where students choose to travel is associated with their drinking behaviors, with students studying in European countries and Australia drinking more heavily than students studying in other countries. Gender is also important as longitudinal studies show increases in drinking for both genders but greater drinking abroad for men [11, 72]. Furthermore, those with more socially motived reasons for drinking and those who may be drinking to cope (e.g., to alleviate feelings of homesickness or feeling out of place) tend to drink at greater levels abroad [10, 65], and those students who believe alcohol will play a large role in their study abroad experience report more alcohol-related consequences while abroad [9].

### The present study

Expanding on our promising pilot findings, we designed the present study to refine and further test the PNF + SAF intervention in five specific ways with a large sample of students across multiple diverse colleges and universities in the US. First, discussions with users of the pilot program indicated that the text-heavy feedback presented was too broad and difficult to absorb. Thus, we will update the normative content to reflect drinking in one’s specific country of study and make the SAF more interactive, including video testimonials from study abroad students discussing engaging in the culture and staying safe abroad. Second, given emerging research highlighting increased risk for study abroad alcohol-related risky sexual behavior and sexual victimization [24–26], we will include content on preventing sexual risk and evaluate sexual risk outcomes. Third, we will expand on the small pilot study to test the refined brief, online preventive approach with a proposed 1200 students on a large scale across multiple universities and colleges (i.e., 50+ institutions that have expressed interest in helping us with recruitment of their students). Fourth, we will examine effects on heavy drinking and consequences during both the high-risk event as well as when students return, as these students are at risk for increased drinking post-return compared to their non-study abroad peers [10, 66, 73]. Finally, we will test mediators of changes in perceived norms and reports of sojourner adjustment abroad (the mechanisms targeted in the intervention) and moderators of intervention efficacy known to be associated with problematic use abroad. Mainly, we will test whether younger students (i.e., those under age 21), men, those studying in Europe, those who drink for social and coping reasons abroad, those with expectancies that alcohol will play a large role in their experience while abroad, and baseline heavier drinkers will benefit most from the PNF + SAF intervention [9–11, 52, 65, 72]. We will also examine history of sexual violence prior to abroad as a moderator of intervention effects on sexual violence outcomes, given prior work documenting that sexual violence history moderates outcomes in online-delivered alcohol and sexual violence programming [74, 75].

## Methods/design

### Overview

The present study will occur in two phases. Phase 1 will involve a large-scale documentation of the drinking patterns of a proposed 2500 students studying abroad and the video collection of testimonials/advice from a proposed 20 students who have previously studied abroad. Normative drinking content and video footage from study abroad students will be used to develop the intervention. In Phase 2, we will conduct a parallel group randomized controlled trial (RCT), where we will test the enhanced intervention among a different sample of 1200 students from approximately 50 US institutions. We will test the efficacy of the intervention on preventing problematic drinking behavior, risky sexual behaviors, and experiences of sexual violence in this high-risk environment during the first and last month abroad and on sustained effects once students return to campus at 1- month and 3- months post-return. The hypotheses are that those student participants who receive the intervention will drink less, experience fewer alcohol-related consequences, engage in fewer risky sexual behaviors, and experience lower rates of sexual violence abroad and back home on campus compared to student participants in a control group.

### Participants

Students will be deemed eligible to participate if they (1) are between the ages of 18 and 24, (2) are signed up to study abroad in one of the 12 most popular destinations (i.e., United Kingdom, Italy, Spain, France, Germany, China, Ireland, Australia, Costa Rica, Japan, South Africa, and Mexico; representing 60% of study abroad students [1]), and (3) plan to study abroad for between 8 and 20 weeks (approximately one quarter/semester), which represents about two-thirds of all student abroad students [1].

### Procedures

Representatives from the study abroad offices at the 50 institutions will email prospective students to inform them about the study. Interested students will fill out an online sign-up sheet to assess eligibility. We will then randomly invite students into the study to ensure we have adequate numbers of both male and female genders represented. Invited students can consent to participate in the study and fill out measures collected online. Participants’ confidential data will be tracked by PIN codes. Participants will read an electronic IRB consent form and indicate consent before enrolling in the study. We will recruit 1200 participants who will be randomly assigned to the intervention condition (N = 600) or a control condition (N = 600). Intervention participants will receive the 30 to 40 min intervention as described below. Control participants will receive a link to a general website offering study abroad advice and will be asked to spend at least 30 to 40 min reviewing their institution’s study abroad website content, including policies for drinking abroad. This control condition was selected as a form of “treatment as usual” as our conversations with study abroad personnel indicated this is the extent of typical information students receive about alcohol use abroad. Many students also attend predeparture informational orientation sessions where alcohol use and sexual risk may or may not be briefly discussed; we will control for receipt of orientation sessions that cover such topics in analyses.

**Fig. 1:**
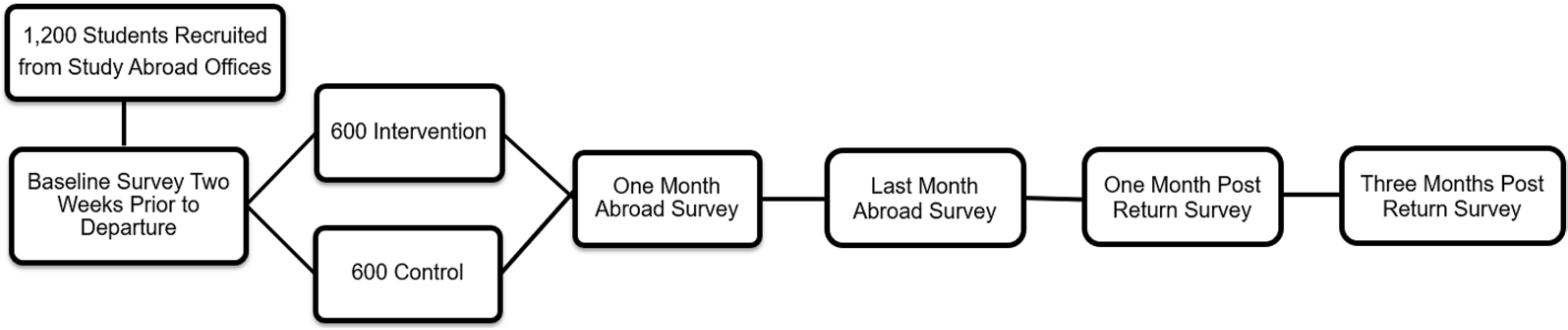
Phase 2 randomized controlled trial study flow

**Fig. 2:**
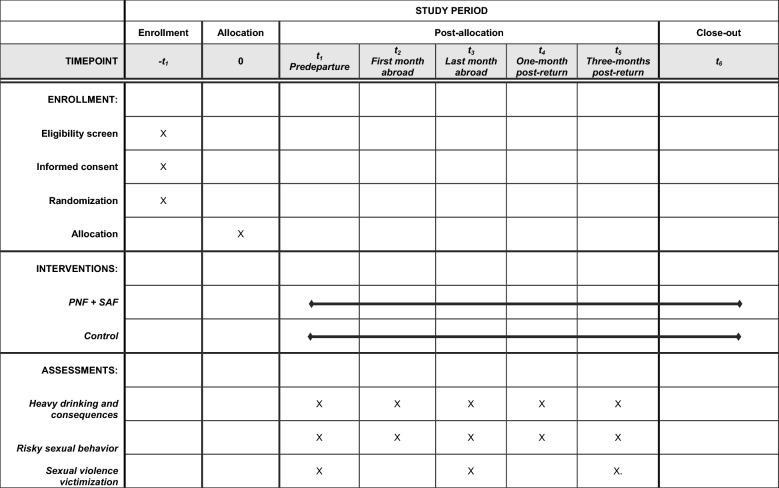
SPIRIT flow diagram of the RCT schedule of enrollment, interventions, and assessments

Participants will complete a survey approximately 2 weeks prior to departure, one survey during their first month abroad, one during their last month abroad, one during their first month back in the US, and a final follow up 3 months post-return to the US. The last survey completed abroad represents the final 30 days of the trip, which, for the majority of students in the study, will be the third month abroad. Length of program will be controlled for in analyses. Participants will receive a $20 Amazon gift card for each of these 15 to 20 min surveys that they complete. Email, text, and phone call reminders will assist with reducing attrition on surveys, which helped us retain 80% of participants in our pilot work and other RCTs [52, 76]. A diagram of participant flow through the intervention study is found in Fig. 1. Figure 2 contains a SPIRIT (Standard Protocol Items: Recommendations for Interventional Trials) flow diagram of the RCT schedule of enrollment, interventions, and assessments.

### Phase 1 documentation of drinking norms and student testimonials

In the first phase of this project, we collected normative drinking data from 2650 study abroad students from 65 universities and colleges. Normative drinking by gender and by country will be presented in the intervention. Based on our prior work, we expected norms to be generally moderate and they will be displayed in a manner that documents that the majority of students abroad drink moderately rather than heavily. The PNF serves to correct misperceived norms and participants see that their intended drinking at predeparture, as well as their perceptions of peer drinking, may be more than the actual drinking of their peers abroad. In addition, we proposed to interview study abroad students about their experiences with drinking (both by peers and by local people) and experiences with sojourner adjustment abroad. Selected clips from these interviews will be loaded into the intervention template so RCT student participants can hear about moderate drinking abroad, such as how local people view alcohol as a complement to a meal rather than as a means to get intoxicated, and how peers that spent most of their time in bars with other Americans risk missing out on meaningful social and cultural experiences.

Video clips will also display students discussing ways in which they engaged with the culture while abroad, with content focused on facets of sojourner adjustment [77]. For example, clips will show students discussing tips and strategies for how they started conversations with local people, how they found out about local cultural events, what they did to help themselves feel more like a local person rather than a tourist, and how they overcame anxiety about using the local language and strengthened their cultural experience by practicing with local people. Negative sojourner adjustment will also be addressed, with clips of students discussing how they expanded beyond their American student network while abroad and how they managed feelings of homesickness and “culture shock” while abroad. Lastly, video clips will feature students describing ways in which they stayed safe while abroad, with an emphasis on prevention of sexual violence victimization abroad. Students will discuss avoiding unwanted sexual experiences, tips for protecting oneself if choosing to have a sexual relationship abroad, and seeking help if one does experience any sexual violence abroad.

### Phase 2 RCT

A sample of 1200 eligible students will be randomly assigned via computer-generated random numbers to complete the intervention (N = 600) or control (N = 600). Participants will be randomly selected from the interested students for inclusion and randomized to the intervention or control conditions in a stratified fashion to ensure equal cells by gender (male, female), age (21+ and under 21), host country (European vs. non-European), and institution type (small college vs. large university). If randomized to the intervention condition, participants will immediately receive the intervention after completing the predeparture survey. Participants can view their feedback again if they desire and will be resent a link to their feedback via email during their first and last month abroad.

#### PNF component

**Fig. 3:**
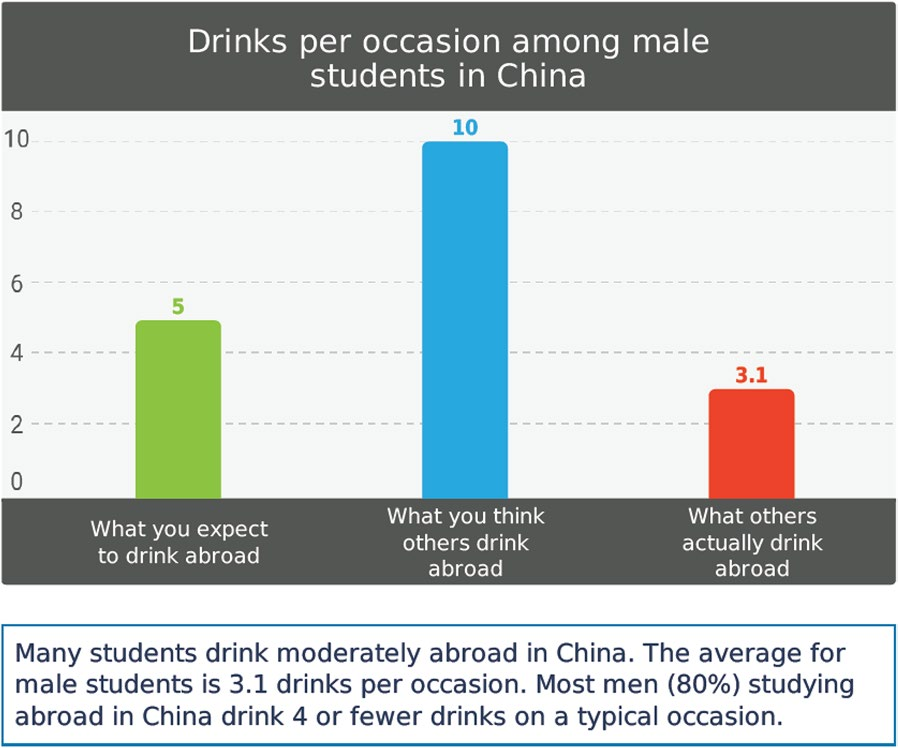
Example graph of PNF for a male student studying abroad in China

During the initial part of the intervention, participants will be asked to indicate how many drinks on average they think they will drink on a typical drinking day while abroad, as well as how many days in a typical month while abroad they will drink 5 or more for males/4 or more for females on an occasion (heavy drinking days). On-screen PNF will be presented containing information on the drinking behavior and attitudes about their gender- and country-specific study abroad peers in graphical and text format. As is standard in PNF interventions [78], a graph will display the participant’s intended drinking, their perception of the drinking of their gender- and country-specific peers, and the actual norm we have collected in Phase 1 (see example in Fig. 3). Text will also display moderate drinking norms (e.g., males students in China report drinking about 3 drinks per occasion”). This PNF format has been used in our prior work and with other event-specific prevention interventions targeting intended drinking behavior prior to high-risk events such as 21st birthday and spring break trips [41, 49, 50]. Participants will then watch video clips of students discussing that drinking among both peers and local people was moderate and less than they had expected before they went abroad.

#### SAF component

The SAF-focused content of the intervention will be similar to what we used in our pilot work [52], which was developed through a thorough review of the literature on sojourner adjustment [55, 60, 77] and focus groups with students to generate tips and strategies for meeting goals of sojourner adjustment abroad. As noted above, we will enhance the SAF by making it more interactive through the use of video testimonials [77]. Participants will view text-based tips and strategies and watch clips of students discussing how they engaged with the culture while abroad. All content focuses around the four aspects of positive sojourner adjustment (social interaction with host nationals, cultural understanding and participation, language development and use, host culture identification) and the two negative sojourner adjustment factors while abroad (i.e., social interaction with co-nationals [(i.e., other Americans], homesickness/feeling out of place) [77]. These video testimonials were collected from eight students that had recently returned from study abroad experiences in several different countries. Students were selected based on diversity of race/ethnicity, gender, and location of study abroad. They were interviewed on campus using a structured interview based on the facets of sojourner adjustment [77]. Clips from these interviews were vetted with study abroad experts and selected based on sufficient coverage of the cultural engagement issues students may encounter while abroad.

#### Staying safe component

Based on review evidence-based approaches for sexual violence prevention back on campus [74, 75], we will include components to address safety behaviors while abroad in three areas: avoiding unwanted sexual experiences abroad, having a safe sexual relationship abroad, and seeking help if one experiences sexual violence or a negative sexual consequence abroad (e.g., contracting a sexually transmitted disease). The intervention will contain text-based tips and protective strategies to be used abroad to reduce sexual risk and sexual violence victimization, as well as clips of students discussing these tips and strategies and encouraging help seeking if one should experience sexual violence abroad. Strategies include having a plan to get out of a potentially unsafe situation, using protection if choosing to engage in sexual activity, ensuring that consent is received during sexual experiences, and looking out for friends and stepping in if they appear to be in a dangerous situation (bystander intervention). Video clips of students discussing methods they used to stay safe abroad will be included. All included content was vetted with research and clinical experts in sexual violence.

## Analytic plan

### Main effects of the PNF intervention

We will analyze five outcomes (total drinks per week, number of heavy drinking days, alcohol-related consequences, risky sex behaviors, and experience of any sexual violence victimization) within a multilevel modeling framework, with the level 1 units being measurement occasions, the level 2 units being individuals, and the level 3 units being schools. Our models will include random effects for the individual students as well as schools to properly adjust for correlations among the repeated measures within an individual student and the students attending the same US school. By analyzing within a multilevel framework, we can investigate whether the intervention is differentially effective at different time points. The main analysis is to estimate the effect of the intervention compared to control at each follow-up time (the first and last month abroad and 1- and 3- months post-return). The N of 1200 was determined in a priori power analyses to be sufficient to detect small to moderate intervention effect sizes for all primary outcomes.

#### Outcome measures

Primary outcomes of the intervention are prevention of heavy and problematic drinking abroad, prevention of risky sexual behaviors abroad, and prevention of any sexual violence victimization abroad. Any observed intervention effects seen during abroad on the first and last month abroad surveys are anticipated to sustain through the first- and third-month post-return to the US, such that intervention participants will drink less, experience fewer problems, engage in fewer risky sexual behaviors, and experience fewer sexual violence events than control participants in the 3 months following return from abroad.

##### Heavy drinking and alcohol-related consequences

Three prespecified drinking outcomes are total drinks per week, number of heavy drinking occasions, and number of alcohol-related consequences in the past 30 days. Drinking in the past 30 days will be assessed at all time points with the Daily Drinking Questionnaire (DDQ; [79]), which asks participants about their typical drinking on each day of a typical week in the past month. It will be used to generate the main drinking outcome of drinks per week but will also yield drinking variable we will look at descriptively (typical drinks consumed per occasion, drinking days per week). A single item will also assess frequency of heavy drinking (5+ drinks on one occasion for males; 4+ drinks on one occasion for females; known in some studies as “binge drinking”). Consequences from alcohol use in the past month will be assessed on all surveys with the 24-item Brief Young Adult Alcohol Consequences Questionnaire (B-YAACQ), a measure of negative consequences often used with the college population [80, 81]. The scale assesses alcohol-related consequences experienced in the past month (yes/no) with a sum score from 0 to 24.

##### Risky sex

Risky sex (e.g., number of partners, use of condoms) in the past month will be assessed with the Sexual Risk Survey (SRS), a 23-item measure designed and validated with college student samples [82]. The SRS will be modified to assess past month behavior as opposed to past 6-month behavior so we can assess this outcome during equal time periods across surveys. The SRS allows for open-ended responses that are then recoded into ordinal categories for generating an overall score, as well as five composite scores of sexual risk taking with uncommitted partners, risky sex acts, impulsive sexual behaviors, intent to engage in risky sexual behavior, and risky anal sex acts [83]. A composite SRS score will be used in analyses as the risky sex outcome, with specific subscale scores examined descriptively.

##### Sexual violence victimization

Sexual violence victimization will be assessed primarily with the Sexual Experiences Survey (SES) [84–86], validated with college students and used in prior work with study abroad students [16, 17, 25]. The measure will be modified and informed by items from the National Drug-Facilitated, Incapacitated, and Forcible Rape study [87] to include 12 gender- and sex-specific items that reflect five aspects of sexual violence victimization: non-consensual/unwanted sexual contact, sexual coercion, completed sexual assault by force, alcohol- and drug-facilitated sexual assaults, and attempted sexual assault, as well as a composite score of any type of sexual violence victimization. The specific items and gender- and sex-specific wording are included in the Appendix. Response options at predeparture capture whether any sexual violence victimization was experienced prior to going abroad (both in college and prior to college). Participants will fill out the measure again during the last month abroad survey to reflect experience of any sexual violence victimization during the entire trip abroad. Finally, participants will complete the measure again during the 3-month post-return surveys to reflect whether they have experienced any sexual violence victimization since returning to the US from study abroad. Experience of any sexual violence abroad will be the main outcome for sexual violence victimization, but we will decribe the categories of non-consensual/unwanted sexual contact, sexual coercion, completed sexual assault by force, alcohol- and drug-facilitated sexual assaults, and attempted sexual assault descriptively.

### Mediator and moderator effects on the intervention

We will investigate the mechanisms of effects through mediation (changes in perceived norms; experience with sojourner adjustment). Mediation will be investigated within the multilevel modeling framework, using bootstrapping to investigate the standard errors (and hence statistical significance) of mediated effects [88–91]. Second, we will investigate potential moderators (age, gender, host country, institution type, race/ethnicity, baseline drinking, drinking motives while abroad, predeparture expectancies, history of sexual violence) of the association between the intervention and main outcomes by incorporating multiplicative interaction terms into the model described above. Given the high number of candidate moderators in our analyses, we will adjust for multiple testing concerns in our moderation analyses by using a Benjamini–Hochberg adjustment to the *p*-value.

#### Measures of mediators

The intervention has two major components (PNF and SAF), and we have included measures to assess whether changes in perceptions and engagement in positive sojourner adjustment mediate observed effects of the intervention.

##### Perceptions

Using a modified Drinking Norms Rating Form (DNRF) [92] participants will be asked about perceived drinking of a typical gender-specific study abroad student in their host country and of a typical native in their host country (i.e. someone born in the country and living there currently). The DNRF is modeled after the DDQ to assess perceptions for each reference group regarding perceived total drinks per week, perceived drinking days, and perceived average drinks during a typical month during the same time period the participants is studying abroad. Perceived number of heavy drinking days per month will also be assessed. Changes in these perceptions (average drinks per occasion, heavy drinking days) are hypothesized to serve as mediators of the intervention as these two perceptions will be directly targeted in the PNF.

##### Sojourner adjustment

We will use the 24-item Sojourner Adjustment Measure (SAM; [77]) to assess the four positive and two negative sojourner adjustment factors. This scale contains a 7-point Likert scale (1 = disagree strongly to 7 = agree strongly) to assess engagement in the four factors of positive sojourner adjustment and two negative sojourner adjustment factors. The SAM will assess actual experience of the six factors abroad to examine changes in sojourner adjustment over time as a mediator of intervention effects. Each of the six factors will be included separately in analyses: the four positive factors of social interaction with host nationals, cultural understanding and participation, language development and use, host culture identification; the two negative factors of social interaction with co-nationals and homesickness/feeling out of place. The SAM factors have demonstrated adequate reliability and convergent validity with established measures of acculturation [77].

#### Measures of moderators

Based on prior work and pilot study findings, we hypothesize that younger students (i.e., those under age 21), men, those studying in Europe, those who drink for social and coping reasons abroad, those with expectancies that alcohol will play a large role in their experience while abroad, and baseline heavier drinkers will benefit most from the PNF + SAF intervention [9, 11, 52, 72]. Items on the predeparture survey will assess age, gender, and location of study. Drinking motives will be assessed in the two abroad assessments with two subscales from the 20-item Drinking Motives Questionnaire–Revised [93] regarding coping and social motives (5 items each). This measure has displayed adequate reliability in general student and study abroad student samples [10, 94, 95]. Expectancies about the role alcohol will play in the study abroad experience will be assessed at predeparture with a measure we have used in our study abroad work (α = 0.84 in prior work) [9]. Participants will rate their agreement with 13 statements (e.g., “I will drink alcohol more often abroad than I drink now,” “alcohol will make my study abroad experience more fun”) from 0 (strongly disagree) to 4 (strongly agree). Baseline heavy drinking will be defined as a continuous value for total drinks per week from the DDQ. History of sexual violence will be defined as any experience of sexual violence prior to abroad (either in college or prior to college). Institution type (small college/university vs large university) and student race/ethnicity will also be accessed and be examined as additional exploratory moderators.

## Discussion

### Limitations and alternative methods considered

We have considered alternative methodological approaches and attempted to address limitations to this study protocol. First, the use of longitudinal self-report measures collected via the Internet could be associated with attrition and bias. However, research suggests confidential surveys enhance reliability and that validity and response rates are higher for web than mailed surveys [96–99]. In addition, we achieved retention rates of 80% or better in the pilot study with multiple assessments while abroad [52].

Second, we considered potential iatrogenic effects from the PNF, but note that studies with college students have suggested that iatrogenic effects are rare in PNF interventions [40, 41]. Light drinkers and non-drinkers who receive PNF report less drinking and more sustained abstinence compared to non-PNF control participants [100]. The manner in which we frame our norms presentation also minimizes iatrogenic effects. For example, it may be accurate to state “only 1 in 10 students studying in Ireland abstained during their trip,” yet this message conveys that abstaining is actually atypical and *only* a few students do it. Instead, we would combine this information with moderate drinkers and frame the message as “80% of students studying in Ireland report drinking less than 3 drinks on a typical night,” which conveys that moderate drinking is typical. This is how we framed normative drinking messages in our pilot work [52].

Third, we chose three primary drinking outcomes because it allows us to see nuanced heavy and problematic behavior. Frequency may increase as a function of the culture abroad (e.g., wine with dinner) and may not be particularly problematic; thus heavy drinking in the form of total weekly quantity and frequency (total weekly drinks) and heavy drinking (i.e., drinking enough to be intoxicated; binge drinking), as well as number of consequences, are our outcomes of interest. We also focus on 12 locations to allow us to personalize the content. These common host countries are skewed European; however, these sites represent 60% of all students abroad [1] and we do include representation of the most popular Asian and Latin American host countries. If the approach is successful, country-specific content for the less popular sites abroad can be developed.

Lastly, we primarily focus on sexual assault victimization and not perpetration as we will likely be underpowered to report any meaningful effects on the low base rate of admitted perpetration in the sample. Both men and women report sexual risk abroad [9] and we include gender as a moderator in outcome analyses.

## Conclusion

In sum, the proposed RCT innovatively extends normative feedback interventions by examining a tailored PNF-adapted intervention that uses additional content regarding SAF to reduce alcohol and sexual risk in young adults studying abroad—a significant and understudied high-risk group entering into a risky foreign context. Although study abroad and student affairs personnel recognize drinking abroad and sexual risk as major concerns and desire empirically-based programs for students as they enter a known period of risk, there currently are few targeted resources for this population. If the proposed intervention is efficacious, as hypothesized, it can be widely implemented at institutions across the country to help prevent the problematic drinking behavior abroad that negatively affects students and institutions, as well as serve as a model to address alcohol risk for other young adults living and traveling abroad.

## Data Availability

Once collected, deidentified data from this study will be available by request to the corresponding author 1 year after all aims of the project are completed. Requestors of data will be asked to complete a data-sharing agreement that provides for (1) a commitment to using the data only for research purposes and not to identify any individual participant; (2) a commitment to securing the data using appropriate computer technology and (3) a commitment to destroying or returning the data after analyses are completed.

## Appendix

Modified sexual experiences survey reflecting study abroad context and incorporating items from the National Drug-facilitated, Incapacitated, and Forcible Rape Study

**Table:**

Domain	Female item wording	Male item wording	Gender-neutral item wording
Introduction	Intro to items: Many women have experienced unwanted sexual advances at some point during their lives. Women do not always report such experiences to police or discuss them with family or friends. Such experiences can happen anytime in a woman’s life—even as a child. The person making these unwanted advances can be friends, boyfriends, girlfriends, coworkers, teaching assistants, supervisors, family members, strangers, or someone they just met. The person making the unwanted sexual advances can be male or female	Intro to items: Many men have experienced unwanted sexual advances at some point during their lives. Men do not always report such experiences to police or discuss them with family or friends. Such experiences can happen anytime in man’s life—even as a child. The person making these unwanted advances can be friends, girlfriends, boyfriends, coworkers, teaching assistants, supervisors, family members, strangers, or someone they just met. The person making the unwanted sexual advances can be male or female	Many people have experienced unwanted sexual advances at some point during their lives. Men and women do not always report such experiences to police or discuss them with family or friends. Such experiences can happen anytime in a person’s life—even as a child. The person making these unwanted advances can be friends, boyfriends, girlfriends, coworkers, teaching assistants, supervisors, family members, strangers, or someone they just met. The person making the unwanted sexual advances can be male or female
Nonconsensual/unwanted sexual contact (1 item)	1. Has anyone ever fondled, kissed, or rubbed up against the private areas of your body (lips, breast/chest, crotch or butt) or removed some of your clothes without your consent *(but did not attempt sexual penetration)*?	1. Has anyone ever fondled, kissed, or rubbed up against the private areas of your body (lips, chest, crotch or butt) or removed some of your clothes without your consent *(but did not attempt sexual penetration)*?	1. Has anyone ever fondled, kissed, or rubbed up against the private areas of your body (lips, breast/chest, crotch or butt) or removed some of your clothes without your consent *(but did not attempt sexual penetration)*?
Sexual coercion (2 items)	2. Has anyone ever had sex with you without your consent by telling lies, threatening to end the relationship, threatening to spread rumors about you, making promises you knew were untrue, or continually verbally pressuring you after you said you didn’t want to. By having sex, we mean that a man or boy put his penis in your vagina, your anus, or your mouth 3. Has anyone ever had sex with you without your consent by showing displeasure, criticizing your sexuality or attractiveness, getting angry but not using physical force, after you said you didn’t want to. By having sex, we mean that a man or boy put his penis in your vagina, your anus, or your mouth	2. Has anyone ever had sex with you without your consent by telling lies, threatening to end the relationship, threatening to spread rumors about you, making promises you knew were untrue, or continually verbally pressuring you after you said you didn’t want to. By having sex, we mean that a man or boy put his penis in your anus or your mouth, someone made you put your penis in their vagina or anus, or someone made you put your mouth on their vagina or anus 3. Has anyone ever had sex with you without your consent by showing displeasure, criticizing your sexuality or attractiveness, getting angry but not using physical force, after you said you didn’t want to. By having sex, we mean that a man or boy put his penis in your anus or your mouth, someone made you put your penis in their vagina or anus, or someone made you put your mouth on their vagina or anus	2. Has anyone ever had sex with you without your consent by telling lies, threatening to end the relationship, threatening to spread rumors about you, making promises you knew were untrue, or continually verbally pressuring you after you said you didn’t want to. By having sex, we mean that a man or boy put his penis in your vagina, your anus, or your mouth; someone made you put your penis in their vagina, anus, or mouth; or someone made you put your mouth on their vagina or anus 3. Has anyone ever had sex with you without your consent by showing displeasure, criticizing your sexuality or attractiveness, getting angry but not using physical force, after you said you didn’t want to. By having sex, we mean that a man or boy put his penis in your vagina, your anus or your mouth; someone made you put your penis in their vagina, anus, or mouth, or someone made you put your mouth on their vagina or anus
Completed sexual assault by force (4 items)	4. Has a man or boy ever made you have sex by using force or threatening to harm you or someone close to you? Just so there is no mistake, by having sex, we mean putting a penis in your vagina 5. Has anyone, male or female, ever made you have oral sex by force or threatening to harm you? So there is no mistake, by oral sex, we mean that a man or boy put his penis in your mouth or someone penetrated your vagina or anus with their mouth or tongue 6. Has anyone ever made you have anal sex by force or threatening to harm you? By anal sex, we mean putting their penis in your anus or rectum 7. Has anyone ever put fingers or objects in your vagina or anus against your will by using force or threatening to harm you?	4. Has a woman or girl ever made you have vaginal sex by using force or threatening to harm you or someone close to you? Just so there is no mistake, by vaginal sex, we mean putting your penis in a vagina 5. Has anyone, male or female, ever made you have oral sex by force or threatening to harm you? So there is no mistake, by oral sex, we mean that a man or boy put his penis in your mouth, someone made you put your mouth or tongue in their vagina or anus, or someone penetrated your anus with their mouth or tongue 6. Has anyone ever made you have anal sex by force or threatening to harm you? By anal sex, we mean putting their penis in your anus or rectum or making you put your penis in their anus or rectum 7. Has anyone ever put fingers or objects in your anus against your will by using force or threatening to harm you?	4. Has anyone, male or female, ever made you have vaginal sex by using force or threatening to harm you or someone close to you? Just so there is no mistake, by vaginal sex, we mean putting a penis in a vagina 5. Has anyone, male or female, ever made you have oral sex by force or threatening to harm you? So there is no mistake, by oral sex, we mean that a man or boy put his penis in your mouth, someone made you put your mouth or tongue in their vagina or anus, or someone penetrated your vagina or anus with their mouth or tongue 6. Has anyone ever made you have anal sex by force or threatening to harm you? By anal sex, we mean putting their penis in your anus or rectum or making you put your penis in their anus or rectum 7. Has anyone ever put fingers or objects in your vagina or anus against your will by using force or threatening to harm you?
Alcohol- and drug- facilitated sexual assaults (2 items)	Intro to section. Some women have had sex when they didn’t want to because they were very high, intoxicated, or even passed out because of alcohol or drugs. We would like to ask you about these types of experiences you might have had. Again, we are interested in these experiences regardless of how long ago it happened, who did it, or whether or not it was reported to police 8. Has anyone ever had sex with you when you didn’t want to after you drank so much alcohol that you were very high, drunk, or passed out? By having sex, we mean that a man or boy put his penis in your vagina, your anus, or your mouth 9. Has anyone ever had sex with you when you didn’t want to after they gave you, or you had taken enough drugs to make you very high, intoxicated, or passed out? By having sex, we mean that a man or boy put his penis in your vagina, your anus, or your mouth	Intro to section: Some men have had sex when they didn’t want to because they were very high, intoxicated, or even passed out because of alcohol or drugs. We would like to ask you about these types of experiences you might have had. Again, we are interested in these experiences regardless of how long ago it happened, who did it, or whether or not it was reported to police 8. Has anyone ever had sex with you when you didn’t want to after you drank so much alcohol that you were very high, drunk, or passed out? By having sex, we mean that a man or boy put his penis in your anus or your mouth, someone made you put your penis in their vagina or anus, or someone made you put your mouth on their vagina or anus 9. Has anyone ever had sex with you when you didn’t want to after they gave you, or you had taken enough drugs to make you very high, intoxicated, or passed out? By having sex, we mean that a man or boy put his penis in your anus or your mouth, someone made you put your penis in their vagina or anus, or someone made you put your mouth on their vagina or anus	Intro to section: Some people have had sex when they didn’t want to because they were very high, intoxicated, or even passed out because of alcohol or drugs. We would like to ask you about these types of experiences you might have had. Again, we are interested in these experiences regardless of how long ago it happened, who did it, or whether or not it was reported to police 8. Has anyone ever had sex with you when you didn’t want to after you drank so much alcohol that you were very high, drunk, or passed out? By having sex, we mean that a man or boy put his penis in your vagina, your anus, or your mouth; someone made you put your penis in their vagina or anus, or someone made you put your mouth on their vagina or anus 9. Has anyone ever had sex with you when you didn’t want to after they gave you, or you had taken enough drugs to make you very high, intoxicated, or passed out? By having sex, we mean that a man or boy put his penis in your vagina, your anus, or your mouth; someone made you put your penis in their vagina or anus, or someone made you put your mouth on their vagina or anus
Attempted sexual assaults (3 items)	10. Has anyone ever *attempted* to have vaginal, oral, or anal sex with you when you did not want to or put fingers or objects in your vagina or anus against your will *but was not successful*? 11. When you had been very *high, drunk, or passed out from alcohol,* has anyone ever *attempted* to have vaginal, oral, or anal sex with you or tried to put fingers or objects in your vagina or anus against your will *but was not successful*? 12. When you had been very *high, intoxicated, or passed out from drugs,* has anyone ever *attempted* to have vaginal, oral, or anal sex with you or tried to put fingers or objects in your vagina or anus against your will but was not successful?	10. Has anyone ever *attempted* to have oral, vaginal, or anal sex with you when you did not want to or put fingers or objects in your anus against your will *but was not successful*? 11. When you had been very *high, drunk, or passed out from alcohol,* has anyone ever *attempted* to have oral, vaginal, or anal sex with you or tried to put fingers or objects in your anus against your will *but was not successful*? 12. When you had been very *high, intoxicated, or passed out from drugs,* has anyone ever *attempted* to have oral, vaginal, or anal sex with you or tried to put fingers or objects in your anus against your will but was not successful?	10. Has anyone ever *attempted* to have vaginal, oral, or anal sex with you when you did not want to or put fingers or objects in your vagina or anus against your will *but was not successful*? 11. When you had been very *high, drunk, or passed out from alcohol,* has anyone ever *attempted* to have vaginal, oral, or anal sex with you or tried to put fingers or objects in your vagina or anus against your will *but was not successful*? 12. When you had been very *high, intoxicated, or passed out from drugs,* has anyone ever *attempted* to have vaginal, oral, or anal sex with you or tried to put fingers or objects in your vagina or anus against your will but was not successful?

*Female items* displayed to participants indicating female sex and female gender. *Male items* displayed to participants indicating male sex and male gender. *Gender-neutral items* displayed to participants who choose not to indicate a sex, chose a gender that is different than their sex, who describe their gender as transgender, or who do not identify as male, female, or transgender.

*Response options at predeparture*: Regardless of how long ago it happened or who made the unwanted sexual advances…

- 0 = No, this has never happened to me
- 1 = Yes, this happened to me before I started college
- 2 = Yes, this happened to me during college
- 3 = Yes, this happened to me before college and during college

*Response options at last month abroad*: Consider your entire trip abroad. Regardless of when it happened during your trip or who made the unwanted sexual advances…

- 0 = No
- 1 = Yes

Also, on the last month abroad survey, all items will be prefaced with “During your study abroad trip…”

*Response options at 3-month post-return*: Consider the past 3 months (i.e., the time since you have been back in the US since your study abroad trip ended). Regardless of when during this time it happened or who made the unwanted sexual advances…

- 0 = No, this has not happened to me
- 1 = Yes, this happened to me since I have returned from study abroad

Also, on the 3-month post-return survey, all items will be prefaced with “During the past 3 months since you have been home from your study abroad trip…”

## Abbreviations

B-YAACQ: Brief Young Adult Alcohol Consequences Questionnaire
DDQ: Daily Drinking Questionnaire
DNRF: Drinking Norms Rating Form
PNF: personalized normative feedback
RCT: randomized controlled trial
SAM: Sojourner Adjustment Measure
SAF: sojourner adjustment feedback
SES: Sexual Experiences Survey
SPIRIT: Standard Protocol Items: Recommendations for Interventional Trials
SRS: Sexual Risk Survey
